# From Synthetic Fragments of Endogenous Three-Finger Proteins to Potential Drugs

**DOI:** 10.3389/fphar.2019.00748

**Published:** 2019-07-03

**Authors:** Elena V. Kryukova, Natalia S. Egorova, Denis S. Kudryavtsev, Dmitry S. Lebedev, Ekaterina N. Spirova, Maxim N. Zhmak, Aleksandra I. Garifulina, Igor E. Kasheverov, Yuri N. Utkin, Victor I. Tsetlin

**Affiliations:** ^1^Department of Molecular Neuroimmune Signalling, Shemyakin-Ovchinnikov Institute of Bioorganic Chemistry, Russian Academy of Sciences, Moscow, Russia; ^2^Sechenov First Moscow State Medical University, Institute of Molecular Medicine, Moscow, Russia; ^3^PhysBio of MEPhI, Moscow, Russia

**Keywords:** three-finger proteins, peptide fragments, Ly6 family, nicotinic acetylcholine receptors, nAChRs

## Abstract

The proteins of the Ly6 family have a three-finger folding as snake venom α-neurotoxins, targeting nicotinic acetylcholine receptors (nAChRs), and some of them, like mammalian secreted Ly6/uPAR protein (SLURP1) and membrane-attached Ly-6/neurotoxin (Lynx1), also interact with distinct nAChR subtypes. We believed that synthetic fragments of these endogenous proteins might open new ways for drug design because nAChRs are well-known targets for developing analgesics and drugs against neurodegenerative diseases. Since interaction with nAChRs was earlier shown for synthetic fragments of the α-neurotoxin central loop II, we synthesized a 15-membered fragment of human Lynx1, its form with two Cys residues added at the N- and C-termini and forming a disulfide, as well as similar forms of human SLURP1, SLURP2, and of *Drosophila* sleepless protein (SSS). The IC_50_ values measured in competition with radioiodinated α-bungarotoxin for binding to the membrane-bound *Torpedo californica* nAChR were 4.9 and 7.4 µM for Lynx1 and SSS fragments, but over 300 µM for SLURP1 or SLURP2 fragments. The affinity of these compounds for the α7 nAChR in the rat pituitary tumor-derived cell line GH4C1 was different: 13.1 and 147 µM for SSS and Lynx1 fragments, respectively. In competition for the ligand-binding domain of the α9 nAChR subunit, SSS and Lynx1 fragments had IC_50_ values of about 40 µM, which correlates with the value found for the latter with the rat α9α10 nAChR expressed in the *Xenopus* oocytes. Thus, the activity of these synthetic peptides against muscle-type and α9α10 nAChRs indicates that they may be useful in design of novel myorelaxants and analgesics.

## Introduction

According to proteomic and transcriptomic studies, a dominant family in the venoms of *Elapidae* snakes (cobras, kraits, coral snakes, and some others) are proteins containing about 60–80 amino acid residues, 4–5 disulfide bonds, and having a common type of the compact spatial structure built of three β-structural disulfide-confined loops (Kini and Doley, 2010; Utkin, 2013; Tsetlin, 2015), explaining their common name of “three-finger proteins” (TFPs). The snake venom TFPs greatly differ in their activity: short- and long-chain α-neurotoxins, as well as certain non-conventional neurotoxins are blocking nicotinic acetylcholine receptors (nAChRs) (Tsetlin, 2015), while another group of TFPs is attacking muscarinic acetylcholine receptors (Karlsson et al., 2000). There are toxins inhibiting β-adrenergic and other G-protein coupled receptors (GPCRs) (Blanchet et al., 2017). Among the recently found snake venom neurotoxins are mambalgins blocking the acid-sensitive ion channels (ASICs) (Brzezicki and Zakowicz, 2018). The targets of the snake venom TFPs are not limited by receptors and ion channels: for example, fasciculins from the *Dendroaspis angusticeps* mamba venom inhibit acetylcholinesterase (Karlsson et al., 1984), while cytotoxins, also known as cardiotoxins, do not have a selective target and penetrate the cell membrane (Dubovskii et al., 2013). It is a long history of using venoms as such for medical purposes (Chan et al., 2016; Estevão-Costa et al., 2018). Some individual components, as α-cobratoxin from the cobra venom and batroxobin from jararaca venom, are tested as possible remedies against pain (Gong et al., 2015) and as defibrinogenating agents (Ding et al., 2018), respectively.

There is no X-ray structure for a snake venom neurotoxin in complex with any whole-size nAChR subtype. However, high-resolution X-ray structures of toxins with the nAChR models are available: α-cobratoxin complex (Bourne et al., 2005) with the acetylcholine-binding protein (AChBP), which is an excellent model for the nAChR ligand-binding domain (LBD) (Smit et al., 2001; Ulens et al., 2006), α-bungarotoxin bound to the chimera of AChBP with the LBD of the α7 nAChR (Huang et al., 2013), as well as to the monomeric LBDs of the α1 and α9 nAChR subunits (Dellisanti et al., 2007; Zouridakis et al., 2014). All these structures, as well as earlier data on chemical modification and mutagenesis of α-neurotoxins, show the important role of the neurotoxin central loop II. Indeed, synthetic fragments of the central loop retained partial activity of the starting toxin and one such peptide was used for production of antisera for native toxin neutralization (de la Rosa et al., 2017).

Thus, synthetic fragments of snake venom toxins look promising for drug design. However, our paper is focusing not on the synthetic fragments of α-neurotoxins, but on the Ly6 proteins. Some of these proteins interact with nAChRs [see reviews (Ibañez-Tallon and Nitabach, 2012; Tsetlin, 2015)] and are considered as potential drugs (Miwa and Walz, 2012; Narumoto et al., 2013; Swamynathan et al., 2017; Lyukmanova et al., 2016a). Among them are mammalian proteins Lynx1 attached to the membrane in the vicinity of nAChRs by the glycosyl phosphoinositide anchor, and a secreted water-soluble protein SLURP1 (Durek et al., 2017). In view of the perspectives shown by synthetic fragments of α-neurotoxins, we synthesized the loop II fragments of these proteins and tested their activity against several nAChR subtypes. In case of positive results, the advantage of such peptides would be their origin from the endogenous proteins, allowing to expect from them only minimal toxicity. We synthesized the fragments of human Lynx1, SLURP1, and SLURP2, as well as a fragment of the a central loop of the *Drosophila* protein SSS, because for this protein [promoting sleep in *Drosophila* by inhibiting its neuronal nAChRs and activating a potassium channel (Wu et al., 2014)] the essential role of its whole loop II was demonstrated by heterologous expression of the protein lacking the N- and C-terminal loops (Wu et al., 2016).

## Materials and Methods

### Solid-Phase Peptide Synthesis of the Central Loop Fragments of the Ly6 Proteins Interacting With nAChRs

**Figure 1:**
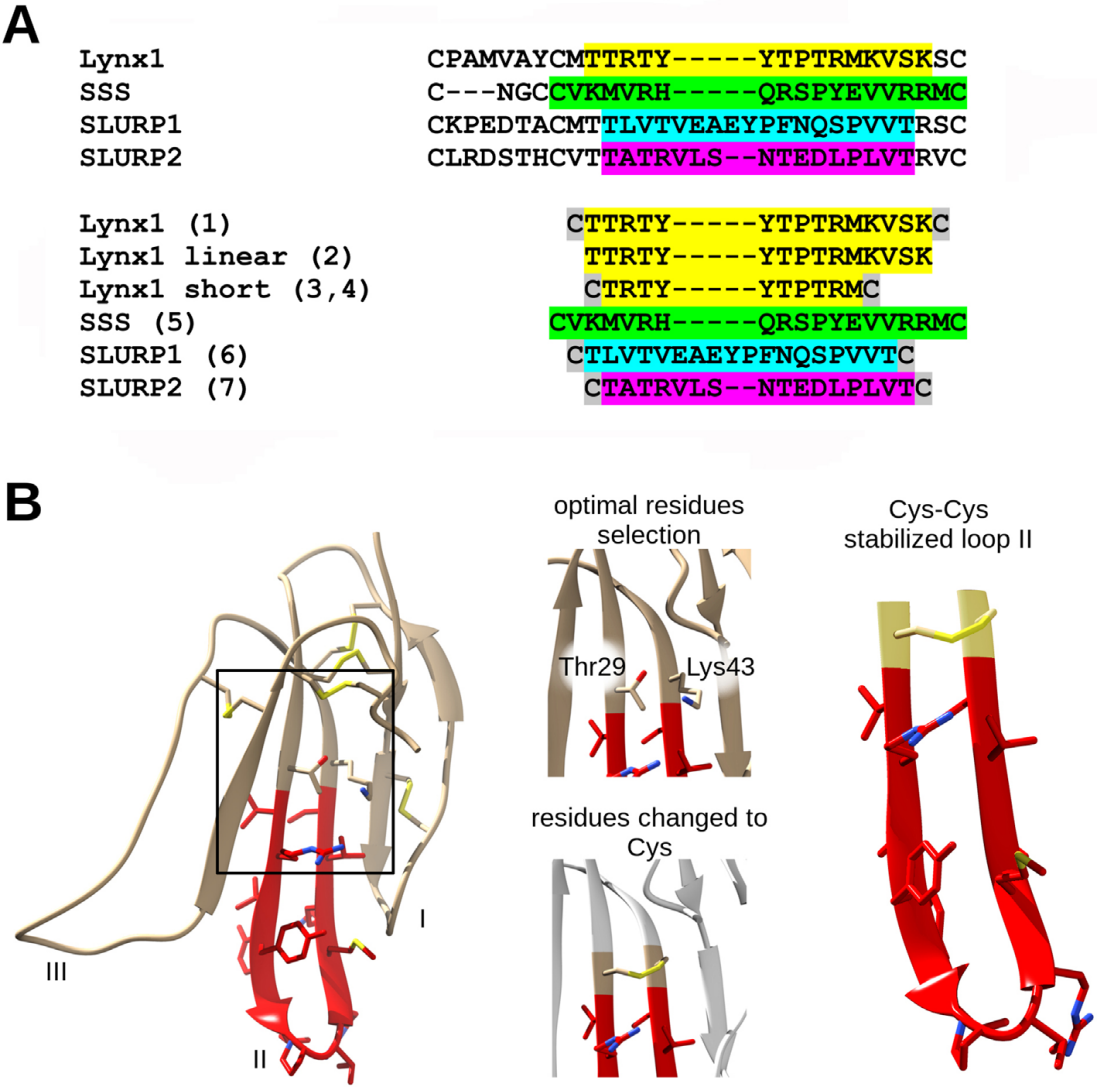
Loop II fragment selection and design. **(A)** Loop II amino acid sequences alignment of Ly6 proteins. The respective synthetic peptide fragments of loop II prepared and studied in present work are labeled with numbers from **1** to **7** (see the Results section) and highlighted with the appropriate color. Cys residues added for the stabilization purposes are highlighted grey. **(B)** Design of artificial disulfide-stabilized loop II mimetics. To achieve hairpin conformation close to native, water-soluble Lynx1 structure (PDB 2L03) has been analyzed, Met28 and Ser44 (in case of peptide **1**) or Thr29 and Lys43 (in case of peptide **3**) residues were chosen because their radicals are situated on the same side of the beta-sheet. Selected residues were interchanged to Cys residues in loop II mimetic peptides. The same logic was applied to SLURP1 and SLURP2 structures. SSS loop II fragment was designed based on the sequence alignment.

The amino-acid sequences of the chosen synthetic fragments and a typical form of the Ly6 protein moiety are presented in **Figure 1**. The peptides were prepared by solid-phase synthesis using Fmoc/t-butyl strategy on tritylchloride-polystyrene resin (Intavis, Cologne, Germany). The disulfides were formed under conventional oxidation conditions: prolonged incubation on air in 50% aqueous acetonitrile at room temperature in the presence N-ethyldiisopropylamine (pH 8.0). All peptides were purified by high-performance liquid chromatography (HPLC) on a 250 × 30 mm C8 column (Dr. Maisch, Ammerbuch-Entringen, Germany) in linear gradient of acetonitrile from 10% to 40% and then lyophilized. The molecular masses determined by electrospray ionization ion trap mass spectrometry (ESI-IT-MS) were very close to theoretically calculated ones (see **Table 1**). The theoretical pI (isoelectric point) computation was done with Compute pI/Mw tool on SIB Bioinformatics Resource Portal (https://web.expasy.org/compute_pi/). The peptides purity (>98%) was confirmed using analytical HPLC. In order to stabilize the spatial structure, Cys residues were added at N- and C-termini to all peptides [except Lynx1 linear (**2**)] and were either left free [as in Lynx1 linear short (**4**)] or formed a disulfide as in Lynx1, Lynx1 short, SSS, SLURP1, and SLURP2 (**1**, **3**, **5**–**7**). In order to prevent formation of disulfides in peptide **4**, it was stored in the lyophilized form and dissolved immediately before the experiment. The structures and characteristics of the synthesized peptides are shown in **Table 1**.

**Table 1:** Structures and characteristics of the synthesized Ly6 peptides.

Ly6 peptide	Peptide sequence	Molecular masses (MH^+^)		pI
		Calculated	Measured	
Lynx1 **(1)**	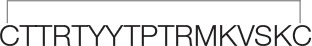	2,036.97	2,036.90	9.70
Lynx1 linear **(2)**	TTRTYYTPTRMKVSK	1,832.96	1,832.85	10.45
Lynx1 short** (3)**	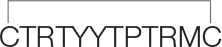	1,493.63	1,493.60	8.90
Lynx1 short linear **(4)**	CTRTYYTPTRMC	1,495.64	1,495.70	8.90
SSS **(5)**	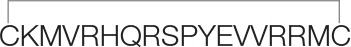	2,278.12	2,278.17	10.09
SLURP1 **(6)**	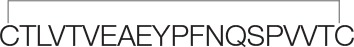	2,200.02	2,200.12	3.80
SLURP2 **(7)**	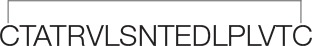	1,935.94	1,936.00	4.37

### Radioligand Analysis of Peptide Interactions With Various nAChR Subtypes, Ligand-Binding Domain of the α9 Subunit and With the AChBP

The binding capacity (IC_50_ values) was estimated in competition with the radioiodinated α-bungarotoxin [prepared as described in Kryukova et al. (2018) with specific radioactivity of 500 Curies/mmol] for association with the membrane-bound *Torpedo californica* nAChR (Hucho et al., 1978) (kindly provided by Prof. F. Hucho, Institute for Chemistry and Biochemistry, Freie Universität Berlin, Germany), human α7 nAChR stably expressed in the rat pituitary tumor-derived cell line GH4C1 (Virginio et al., 2002) (received from Eli Lilly and Company, London, UK), LBD of the human nAChR α9 subunit (Zouridakis et al., 2014) (prepared in the laboratory of Prof. S. Tzartos, Department of Neurobiology, Hellenic Pasteur Institute, Greece), and AChBP from *Aplysia californica* (Lin et al., 2014) (kindly provided by Prof. S. Luo, Key Laboratory for Marine Drugs of Haikou, Hainan University, China).

The suspensions of membranes from *T. californica* ray electric organ (1.25 nM α-bungarotoxin binding sites) or human α7 nAChR expressed in GH4C1 cells (0.4 nM α-bungarotoxin binding sites) or expressed *A. californica* AChBP (150 nM α-bungarotoxin binding sites) were incubated in 50 μl of binding buffer [20 mM Tris-HCl buffer, pH 8.0, containing 1 mg/ml bovine serum albumin (BSA)] for 90 min with various amounts of the Ly6 peptides, followed by an additional 5-min incubation with 0.1–0.2 nM ^125^I-labeled α-bungarotoxin (500 Ci/mmol). The membranes and cell suspensions were applied to glass GF/C filters (Whatman, Maidstone, UK) pretreated with 0.3% polyethylenimine. The samples were then washed (3 × 4 ml) with cold 20 mM Tris-HCl buffer, pH 8.0, containing 0.1 mg/ml BSA and bound radioactivity was measured with a Wallac 1470 Wizard Gamma Counter (PerkinElmer, Waltham, MA, USA). The *A. californica* AChBP samples were applied to two layers of DE-81 filters presoaked in phosphate-buffered saline containing 0.1 mg/ml BSA and washed (3 × 4 ml) with the same buffer. For human α9 LBD, the competition assays were carried out with 100 nM α9 LBD and different amounts of the Ly6 peptides. After incubation, 0.1–0.2 nM ^125^I-labeled α-bungarotoxin (500 Ci/mmol) and 10 μl of Ni^2+^-NTA agarose beads (Qiagen, Hilden, Germany) prewashed twice with binding buffer and diluted three times with the same buffer were added and after additional 5-min incubation (during this time, ^125^I-labeled α-bungarotoxin occupies about 50% of binding sites on the *Torpedo* receptor and about 30–40% on the α7 nAChR), suspensions were applied to glass GF/C filters, washed, and bound radioactivity was measured as described above. Nonspecific ^125^I-α-bungarotoxin binding was determined in the presence of 200-fold excess of α-cobratoxin.

### Two-Electrode Voltage Clamp Analysis of Peptides Interaction With the Rat and Human α9α10, Human α3β2, and Human α4β2 nAChRs

*Xenopus laevis* frogs were fed twice a week and maintained according to supplier recommendations (https://www.enasco.com/page/xen_care).

All experiments were carried out in strict accordance with the World Health Organization’s International Guiding Principles for Biomedical Research Involving Animals. The protocol (protocol number: 251/2018 26.02.18) was approved by Institutional Animal Care and Use Committee basing on the Institutional Policy on the Use of Laboratory Animals of the Shemyakin-Ovchinnikov Institute of Bioorganic Chemistry RAS.

Oocytes were removed from mature, anesthetized *Xenopus laevis* by dissecting abdomen and removing necessary amount of ovarium. Stage V–VI *Xenopus laevis* oocytes were defolliculated with 2 mg/ml collagenase type I (Life Technologies, Camarillo, USA) at room temperature (21–24°C) for 2 h in Ca^2+^-free Barth’s solution composed of (in mM) 88 NaCl, 1.1 KCl, 2.4 NaHCO_3_, 0.8 MgSO_4_, and 15 HEPES-NaOH at pH 7.6. Oocytes were injected with 9.2 ng of rat or human nAChR α9 and α10 cRNA (in a ratio 1:1) or human α3 and β2 or α4 and β2 cRNA (in a ratio 1:1). Oocytes were incubated at 18°C for 2–4 days before electrophysiological recordings in Barth’s solution composed of (in mM) 88 NaCl, 1.1 KCl, 2.4 NaHCO_3_, 0.3 Ca(NO_3_)_2_, 0.4 CaCl_2_, 0.8 MgSO_4_, and 15 HEPES-10NaOH at pH 7.6, supplemented with 40 μg/ml gentamicin and 100 μg/ml ampicillin. Recordings were performed using turbo TEC-03X amplifier (NPI Electronic, Tamm, Germany) and WinWCP recording software (University of Strathclyde, Glasgow, UK). The glass recording electrodes were filled with 3 M KCl and the electrode resistance was 0.1−0.5 MΩ. Membrane potential was clamped at −60 mV. Oocytes were briefly washed with Ba^2+^ Ringer’s solution (Fuchs and Murrow, 1992) composed of (in mM) 115 NaCl, 2.5 KCl, 1.8 BaCl_2_, 10 HEPES at pH 7.2, followed by three applications of 10 μM acetylcholine (ACh). Washout with Ba^2+^ Ringer’s was done for 5 min between ACh applications. Oocytes were preincubated with various concentrations of Ly6 peptides for 5 min followed by their co-application with ACh. To induce ion current, we used ACh in the concentration range from 10 to 30 μM. The peak current amplitudes of ACh-induced responses were measured before (ACh alone) and after the preincubation of oocytes with peptides. The ratio between these two measurements was used to assess the activity of the tested compounds. In the experiments with α3β2 and α4β2, the above technique was also used, except that oocytes were washed in Barth`s solution. All control experiments were performed on the same day.

Rat α9 and α10 cDNAs was cloned in a pGEMHE vector; human α9, α10, α3, and β2 cDNAs were derived from pT7TS vector. Human α4 was cloned in pSP64 vector. Plasmid pT7TS constructs of human nAChR α9 and α10 subunits and human α3 and β2 subunits were linearized with *Xba*I restriction enzymes (NEB, Ipswich, USA). pSP64 vector was linearized with BamH1 restriction enzymes (NEB, Ipswich, USA). Rat α9 and α10 plasmids were linearized using *Nhe*I (NEB, Ipswich, USA). All plasmid constructs were sequencing before mRNA synthesis. The indicated restriction enzymes were chosen as their restriction sites are located downstream of the sequence as it is recommended by the manufacturer in mMESSAGE mMACHINE^®^ High Yield Capped RNA Transcription Kit (Thermo Fisher Scientific, Waltham, USA). mRNAs were transcribed *in vitro* using T7 mMESSAGE mMachine^™^ (Thermo Fisher Scientific, Waltham, USA) and SP6 were prepared using SP6 mMESSAGE mMACHINE^®^ High Yield Capped RNA Transcription Kit (Thermo Fisher Scientific, Waltham, USA). Transcribed mRNA was polyadenylated using the Poly-A-Tailing Kit (Thermo Fisher Scientific, Waltham, USA). The mRNAs were stored up to 6 months at −70°C. Before every use, the degradation levels of mRNAs were checked by gel electrophoresis.

### Statistical Analysis

The binding results were analyzed using ORIGIN 8.0 (OriginLab Corporation, Northampton, MA, USA) fitting to a one-site dose-response curve by the equation: % response  =  100/{1 + ([toxin]/IC_50_)*^n^*}, where IC_50_ is the concentration at which 50% of the binding sites are inhibited and *n* is the Hill coefficient. Data of the radioligand assay are presented as mean with 95% confidence interval (CI) for the indicated number (n) of independent experiments. Paired Student’s t-test was done in ORIGIN 8.0 with the significance level set to p < 0.05.

## Results

**Figure 1** and **Table 1** show that the fragments of several Ly6 proteins were obtained as homogeneous peptides and their structure has been confirmed by mass spectrometry. **Table 1** shows that the synthesized loops of human Lynx1 and *Drosophila* SSS have very close calculated pI values (9.7 and 10.0). They are basic similarly to the respective fragment (with the pI values of 7.9) of a short neurotoxin II [which like Ly6 proteins does not have an additional disulfide in the central loop (Grishin et al., 1973)] and are much closer to pI 10.46 for a weak toxin *Naja kaouthia* respective fragment [which also contains no additional disulfide in the central loop, but similarly to Ly6 proteins possesses additional disulfide in the N-terminal loop (Utkin et al., 2001)]. However, the pI values for SPURP1 and SLURP2 fragments are much lower (3.80 and 4.37, respectively), which might explain a big difference from the Lynx1 and SSS fragments in our biological tests (see below).

### Radioligand Analysis of Ly6 Peptides Interaction With nAChR Subtypes and Their Models

**Figure 2:**
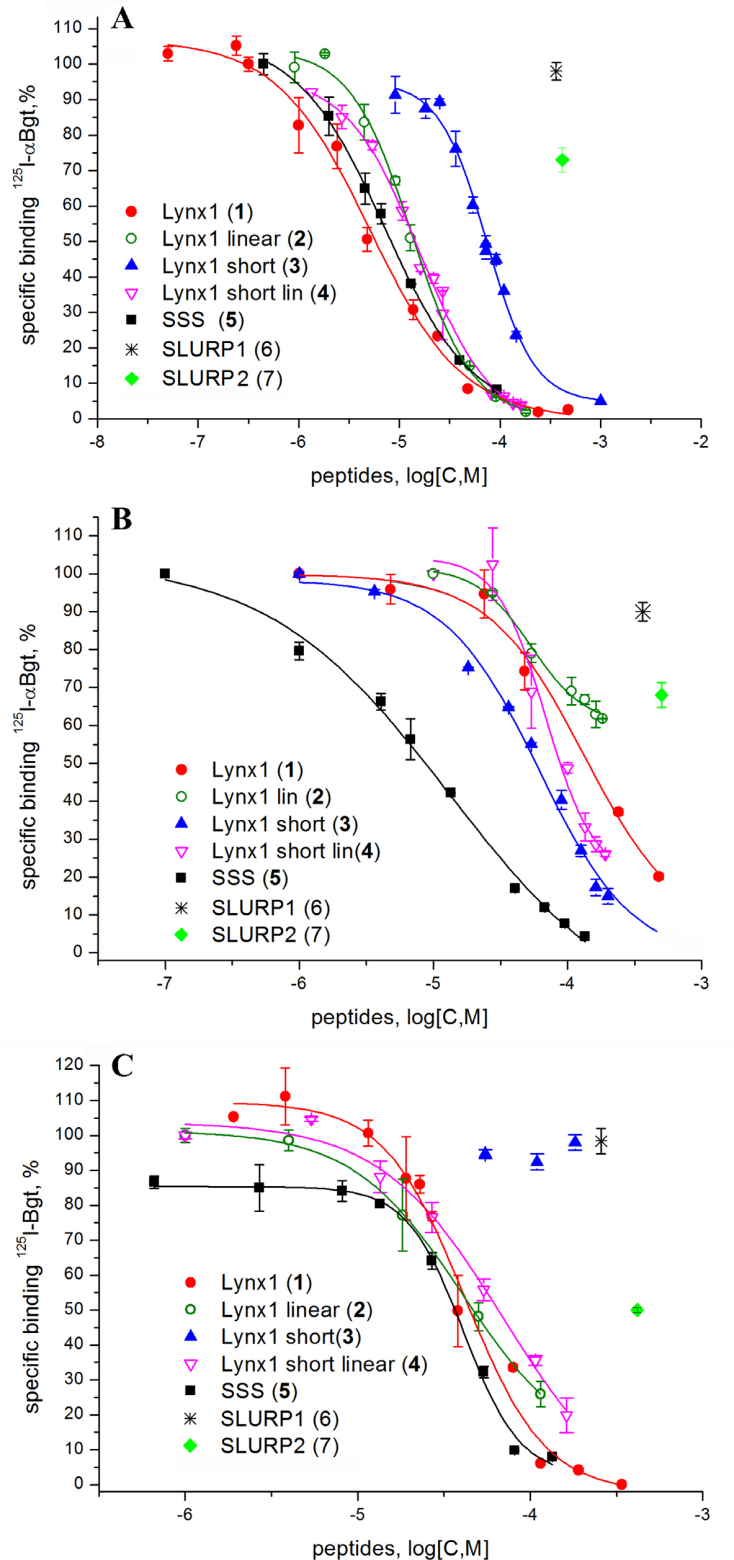
Competition of [^125^I]-labeled αBgt with Lynx1 peptides and SSS fragment for binding to **(A)** *Torpedo californica* nAChR, **(B)** human α7 nAChR expressed in the GH4C1 cell line, and **(C)** ligand-binding domain (LBD) of the human nAChR α9 subunit. IC_50_ values and 95% confidence interval (CI 95%) for these data are presented in **Table 2**. Each data point represents the mean ± SEM of three to four independent experiments.

Since high-affinity binding of radioiodinated α-bungarotoxin (αBgt) to muscle-type, α7 and α9α10 nAChRs occurs at their orthosteric sites, competition with this radioligand allows to detect binding to such sites also for the compounds of interest. **Figure 2** and **Table 2** show that the most active against the muscle-type receptor from *T. californica* was the fragment of Lynx1 (**1**) (IC_50_ = 4.9 µM), but it had almost a 30-fold lower activity against human α7 nAChR. Interestingly, the SSS fragment (**5**) was slightly less active against *Torpedo* receptor (7.4 µM), but 10-fold more active against α7 nAChR than the Lynx1 fragment (**1**) (IC_50_ 13.1 and 147.7 µM, respectively). Although cyclization was expected to stabilize the spatial structure, a linear form of the Lynx1 fragment (**2**) on the *Torpedo* and α7 nAChRs had the activity quite similar to that of the cyclized form. Linear (**4**) and especially cyclic (**3**) shorter forms of Lynx1 fragment were less active against *Torpedo* nAChR, but twofold more active against α7 nAChR than peptide (**1**).

**Table 2:** The binding affinity of synthetic peptides to *T. californica*, human α7 nAChR, human α9 LBD, and *A. californica* AChBP measured by a competitive radioligand assay with ^125^I-α-Bgt. The IC_50_ values and 95% confidence interval (CI) are presented for three to four independent experiments.

Ly6 peptide	IC_50_, μM (95%CI)			
	T. californica	α7	α9 LBD	AChBP
Lynx1 **(1)**	4.9 (3.81–6.30)	173 (42.28–415.7)	41.7 (13.18–52.48)	>100
Lynx1 linear **(2)**	13.2 (11.26–15.68)	>180	40.7 (30.76–53.95)	>100
Lynx1 short **(3)**	74.13 (64.53–84.22)	58.9 (50.19–74.43)	>100	>100
Lynx1 short linear **(4)**	15.60 (13.55–18.62)	69.2 (48.98–98.63)	70.8 (25.7–190.5)	>100
SSS **(5)**	7.4 (7.14–7.73)	13.1 (6.60–26.1)	41.7 (38.19–47.86)	>100
SLURP1 **(6)**	>360	>360	>300	>100
SLURP2 **(7)**	>420	>500	∼400	>100

In competition with ^125^I-αBgt for the binding to α9 LBD, the SSS fragment (**5**), cyclic and linear Lynx1 fragments (**1** and **2**) revealed very similar affinities, their IC_50_ values being around 40 µM. On the other hand, **Table 2** shows that for SLURP1 and SLURP2 fragments even at concentrations over 300 µM, no inhibition of ^125^I-αBgt binding was observed either with muscle and α7 nAChRs, or with α9 LBD. The loss of any inhibition at such high concentrations indicates the absence of nonspecific effects of the studied peptides on the binding of the radioligand to the targets. In fact, SLURP1 and SLURP2 fragments act as scrambled peptides in these experiments. Interestingly, although AChBP is known to bind compounds interacting not only with the nAChRs, but also with different Cys-loop receptors (Sixma and Smit, 2003; Brams et al., 2011) at concentration of about 100 µM, none of the compounds given in **Table 2** competed with ^125^I-αBgt for binding to the *A. californica* AChBP

### Two-Electrode Voltage Clamp Analysis

**Figure 3:**
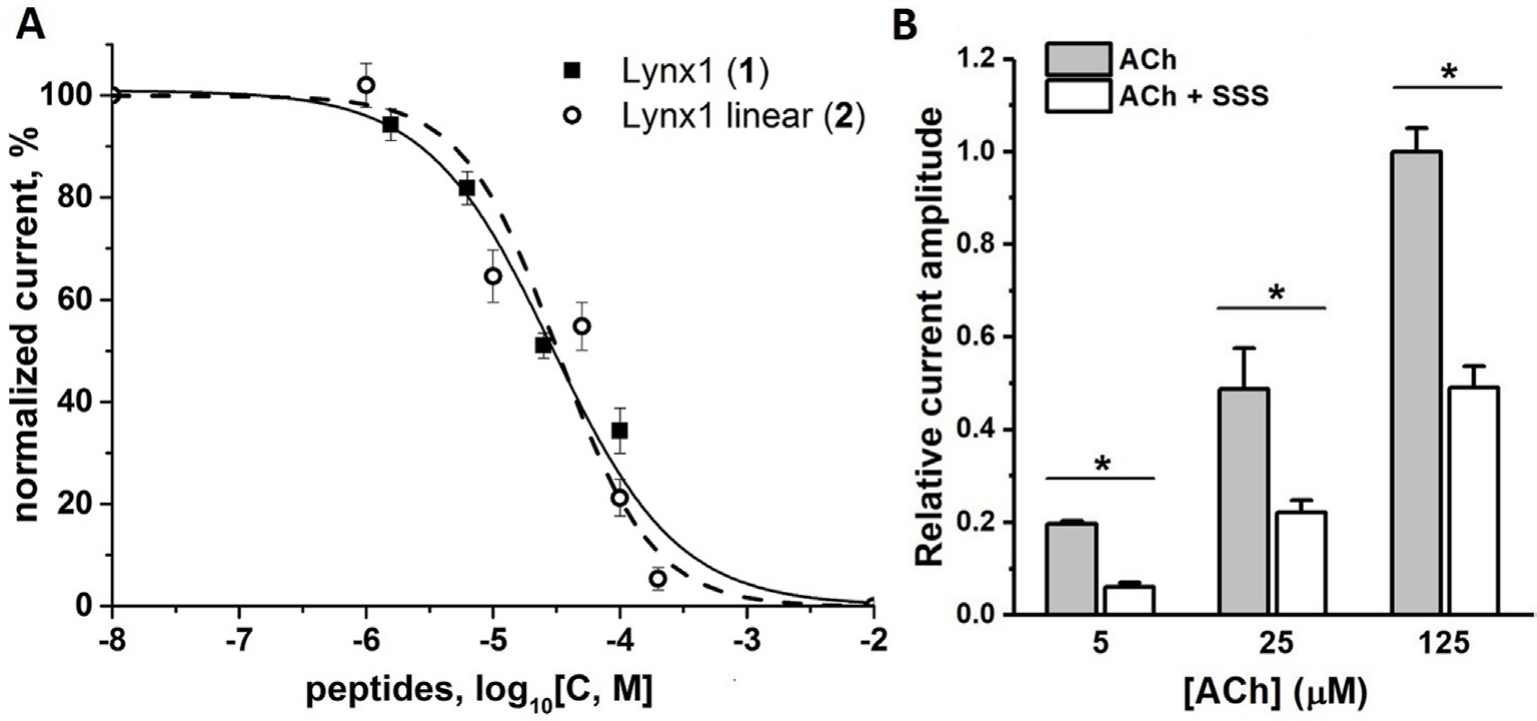
Activity of synthetic Ly6 peptides on human and rat α9α10 nAChRs expressed in *X. laevis* oocytes. **(A)** Inhibition of ion currents induced by 30 μM acetylcholine by Lynx1 **(1)** fragment in the rat (▪, solid line) α9α10 nAChRs or by Lynx1 linear fragment **(2)** in the human (○, dashed line) α9α10 nAChRs. The oocytes were pre-incubated with peptides for 5 min. For Lynx1 **(1)** the calculated IC_50_ was 27.00 μM (CI 95% 14.55–41.26 μM), n = 3. For Lynx1 linear fragment **(2)** the calculated IC_50_ was 32.30 μM (CI 95% 14.70–70.99 μM), n = 3. **(B)** Bar graph of 100 μM SSS fragment **(5)** inhibition of ion currents induced by 25 μM acetylcholine (ACh) in the human α9α10 nAChR. Data are presented as mean ± SEM, n = 3. Paired Student’s t-test, *p* < 0.05 (*black asterisks*, normalized current evoked by acetylcholine in the presence of SSS *vs* normalized current induced by acetylcholine in the absence of SSS).

Since, this work was stimulated by the discovery of SLURP1 inhibition of the human and rat α9α10 nAChRs (Durek et al., 2017), we tested the activity of the synthesized peptides by two-electrode voltage clamp against human α9α10 nAChR expressed in the *Xenopus* oocytes. Unfortunately, no inhibition was registered for the SLURP1 or SLURP2 fragments (**6** and **7**) at concentrations as high as 50 µM (data not shown). On the other hand, although α9α10 nAChR was not detected among the targets of Lynx1 or its water-soluble variant (ws-Lynx1), the Lynx1 fragment (**1**) inhibited rat α9α10 nAChR with IC_50_ of 27 µM (**Figure 3A**). A slightly weaker activity was found for the Lynx1 linear fragment (**2**), while only 50% inhibition was detected for SSS fragment (**5**) at 100 µM concentration (**Figure 3B**).

**Figure 4:**
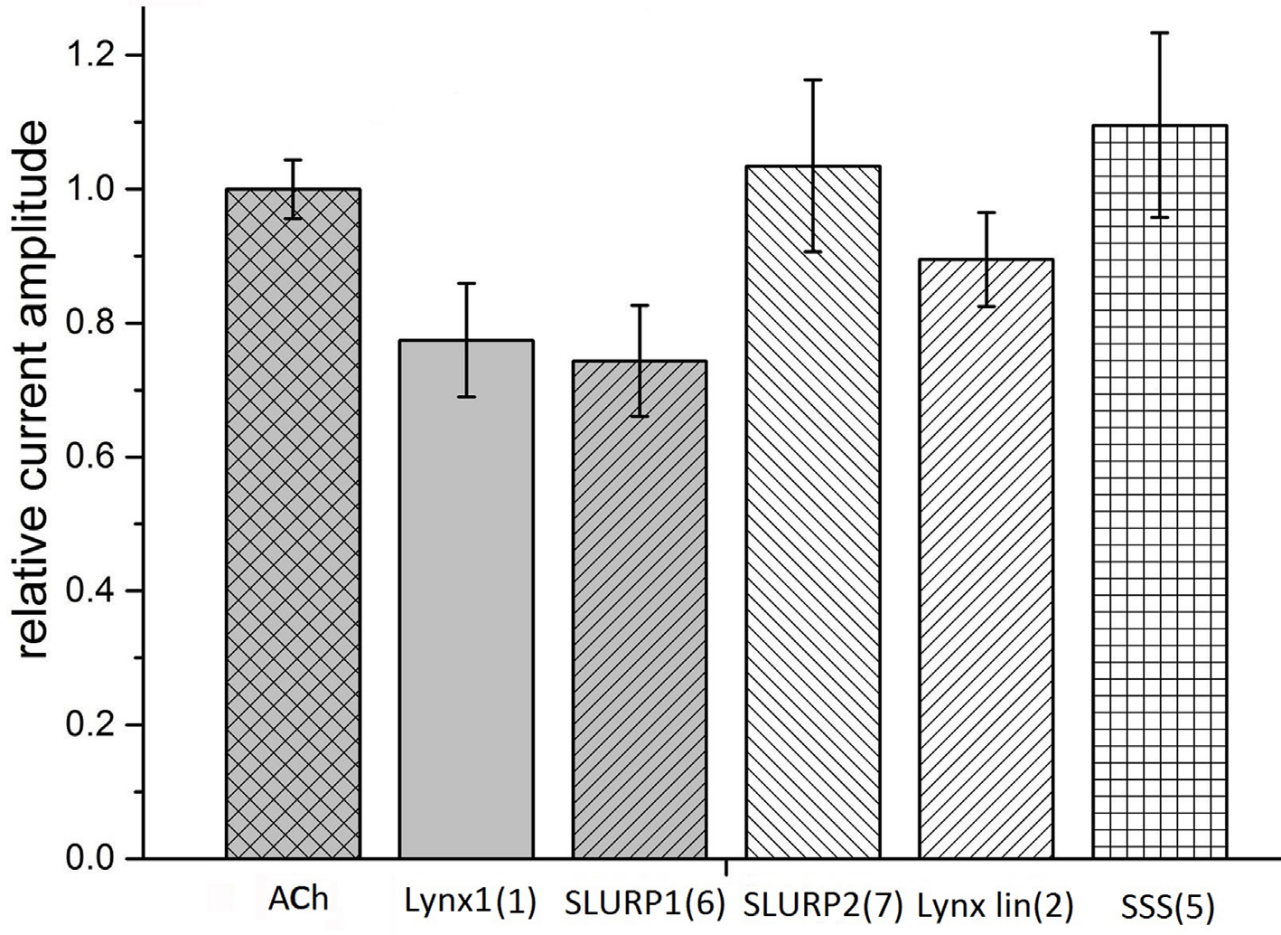
Bar graph of 50 μM Ly6 fragments of Lynx1 **(1)**, SLURP1 **(6)**, SLURP2 **(7)**, Lynx1 linear **(2)**, and SSS **(5)** inhibition of ion currents induced by 30 μM ACh in the human α3β2 nAChR expressed in *X. laevis* oocytes. The current recorded in response to 30 μM ACh application (ACh) is accepted as 1. The current recorded in response to application of acetylcholine in the presence of peptides was compared with the mean between previous and afterwards acetylcholine-induced current. Normalized currents are presented as mean ± SEM, n = 3 [for SLURP2 **(7)**, Lynx1 linear **(2)**, and SSS **(5)**] or n = 5 [for Lynx1 **(1)** and SLURP1 **(6)**]. Ion current values for Lynx1 **(1)** and SLURP1 **(6)** are 0.77 ± 0.08 and 0.74 ± 0.08, respectively.

Taking into account that neuronal αβ heteromeric nAChRs were among the targets of Lynx1 (Miwa et al., 1999; Ibañez-Tallon and Nitabach, 2012), ws-Lynx1 (Lyukmanova et al., 2011, Lyukmanova et al., 2013; Thomsen et al., 2014), and SLURP1 (Durek et al., 2017), we also tested the activity of the synthetic fragments against these receptors in electrophysiological experiments. At 30 µM, Lynx1 fragment (**1**) showed a tendency to inhibition of the current in the α3β2 nAChR by 30% (**Figure 4**). Similar effects were exerted by SLURP1 fragment (**6**) (**Figure 4**), whereas Lynx1 linear fragment (**2**), SLURP2 fragment (**7**), and the SSS fragment (**5**) were inactive. Unfortunately no significant activity of the peptides was detected in comparison to the currents recorded in response to 30 μM acetylcholine application. None of the peptides inhibited the ion currents in α4β2 nAChR (data not shown).

## Discussion

Historically, Lynx-1 was the first protein of the Ly6 family found co-localized in the mouse brain with the α7 and α4β2 nAChRs and shown to inhibit their activity (Miwa et al., 1999). This protein is of special interest to us because several years ago at the Shemyakin-Ovchinnikov Institute a water-soluble analog of Lynx1, devoid of the GPI anchor, was heterologously expressed in *Escherichia coli*. It inhibited ion currents in several nAChR subtypes and its three-dimensional structure was established by ^1^H-NMR (Lyukmanova et al., 2011). Moreover, several mutations in its central loop II were shown to affect the activity (Lyukmanova et al., 2013). However, a water-soluble ws-Lynx1 is only a model of the membrane-bound Lynx1 and the differences in behavior were demonstrated in mouse overexpressing *Lynx 1* genes with or without the sequence coding for the GPI anchor (Miwa and Walz, 2012). The advantage of SLURP1 and SLURP2, the solution structures of which are also known (Lyukmanova et al., 2016b; Vasilyeva et al., 2017), is that they are naturally occurring water-soluble proteins and the research can be done on them as such, rather than on their models. Much information is available about the SLURP1/SLURP2 involvement in several diseases, predominantly in the skin disease Mal de Meleda (Perez and Khachemoune, 2016). However, until recently the work was done not on SLURP1 as such, but on various fusion proteins incorporating SLURP1. One publication revealed potentiation of ion currents in α7 nAChR at nanomolar concentration of the used fusion SLURP1 protein (Chimienti et al., 2003). However, on the human SLURP1, differing from the naturally occurring protein only by the additional N-terminal Met residue, the effect was the opposite: inhibition rather than enhancement of ion current in α7 nAChR and at micromolar, rather than at nanomolar concentration (Lyukmanova et al., 2016b). Moreover, with the SLURP1 prepared by peptide synthesis and having virtually the same primary structure as the native protein, no inhibition of the α7 nAChR took place, but inhibition of several heterooligomeric nAChRs was registered (Durek et al., 2017). Most interestingly, with this SLURP-1 for the first time for an Ly6 protein inhibition of the α9α10 nAChR was observed and binding was proved to occur at an allosteric site (Durek et al., 2017). This finding to a considerable degree stimulated the present work because α9α10 nAChRs are targets for developing new analgesics, as illustrated most convincingly by research on those α-conotoxins, which are selective for this nAChR subtype (Dutertre et al., 2017; Hone and McIntosh, 2018).

As was described in the Introduction, a certain success was achieved in research using mainly the central loop II of three-finger neurotoxins where the structure was additionally stabilized by connecting the N- and C-termini of peptide by the grafted disulfide. We decided to synthesize the appropriate loops of Lynx1, SLURP1, and SLURP 2 and test their possible activities against several nAChR subtypes. We also included the *Drosophila* protein SSS, which affects the sleep processes interacting both with the nAChRs and potassium channels (Wu et al., 2014). Additional importance of this protein for our work, focusing on the central loops II from the TFPs of the Ly6 family, was due to the recently demonstrated functional activity for its heterologically expressed loop II (Wu et al., 2016).

The results in this work show that the synthetic fragments of Lynx1 and SSS demonstrated a low micromolаr binding to the muscle-type *Torpedo* nAChR, as detected in competition with radioactive α-Bgt. Similar binding was registered with the SSS fragment (**5**) in case of the α7 nAChR, while Lynx1 fragment (**1**) was over 10-fold less active. A comparison of the cyclized and linear forms of Lynx1 fragment (**1** and **2**) shows that, contrary to expectations, stabilizing the spatial structure due to connecting the N- and C-termini did not cause a marked increase in the activity against certain targets. Surprisingly, shortening of the peptide (**4**) did not diminish its affinity, as compared to peptide (**2**), indicating that both peptides apparently retain the required conformation. Since peptides still should be relatively flexible it is difficult why cyclization of the short peptide (**3**) is weakening its interaction with the muscle-type and α9/α10 nAChRs.

Both for the SSS and Lynx1 fragments, their association with the α9 LBD was relatively weak (IC_50_ on the average of 40 µM); inhibition (IC_50_) of ion currents in the α9α10 nAChR expressed in *Xenopus* oocytes measured for the Lynx fragment (**1**) was about 30 µM and a slightly less activity was found for the SSS fragment (**5**).

Unfortunately, our hope that the synthetic fragment of SLURP1 will possess at least a part of the activity against α9α10 nAChR found in SLURP1 itself (Durek et al., 2017) was not realized. Neither SLURP1 fragment (**6**) nor that (**7**) of SLURP2 revealed noticeable inhibition of ^125^I-αBgt binding to *Torpedo* and human α7 nAChRs or to the α9 domain. They also did not compete with ^125^I-αBgt for binding to *A. californica* AChBP, but none other synthetic fragment (active against several nAChRs) also manifested a competition. This result is not easy to explain because usually AChBPs are not very discriminative and with low affinity can bind diverse compounds capable of interacting with one or another nAChR subtype or with a different Cys-loop receptor (Brejc et al., 2001; Rucktooa et al., 2009; Brams et al., 2011).

Although our work is focusing on the muscle-type, α7, and α9α10 nAChRs, all of which efficiently bind αBgt (which at nanomolar concentrations also blocks ion currents in them), we checked possible effects of our synthetic fragments on the heterooligomeric α3β2 nAChR. The reason for this is that Lynx1 in the very first publication (Miwa et al., 1999) was shown to inhibit α4β2 nAChRs, while its effects on the function of other heteromeric nAChRs (Ibañez-Tallon and Nitabach, 2012) and on the process of their assembly (Parker et al., 2017) were demonstrated later. In addition, SLURP1 not only inhibited α9α10 nAChR, but also (at low micromolar level) diminished the currents in the α3β2 and α3β4 nAChRs (Durek et al., 2017). The SSS protein was earlier shown to inhibit a heteromeric nAChR of *Drosophila* (Wu et al., 2014). As **Figure 4** shows at least with the α3β2 nAChR, the SLURP1 fragment (**6**) had the activity comparable to that shown by other Ly6 synthetic fragments to one or another target.

It should be mentioned that there are many publications on the activity of the synthetic three-finger toxins and of their fragments: synthetic fragments of the central loop II of α-neurotoxins have been already mentioned (Kasheverov and Tsetlin, 2017), there are also full-size synthetic α-neurotoxins (Rey-Suárez et al., 2012), non-conventional neurotoxins (Poh et al., 2002), TFP toxins inhibiting acetylcholinesterase (fasciculins) (Falkenstein and Peña, 1997), synthetic mambalgins, and their analogs attacking ASICs (Schroeder et al., 2014). Not always in the work on TFP toxins the interest was focused on the central loop: for the synthetic N-terminal loop of cardiotoxin I from the *Naja atra* cobra venom the authors reported antimicrobial activity against Gram-positive and Gram-negative bacteria (Sala et al., 2018). Interestingly, molecular modeling indicates that binding of another cytotoxin I (*Naja mossambica mossambica*) to 20S proteasome (resulting in the inhibition of its chymotryptic activity) is due to the toxin N-terminal loop (Munawar et al., 2015). It is worth to mention that the N-terminal sequence of SIRS (soluble immune response suppressor) has a homology to short α-neurotoxin and its synthetic 21-membered N-terminal fragment was shown to inhibit the development of experimental allergic encephalitis (Webb, 2016).

On the contrary, there is not much work on the synthetic fragments of the Ly6 proteins, and we could not find any publications in scientific literature on the synthetic fragments of those Ly6 proteins which interact with nAChRs or affect their assembly. In the patent database we found one (Walz, 2014) where a series of peptides homologous to Lynx1 fragment (**1**) has been synthesized homologous to Lynx1 fragment (**1**) but devoid of the N- and C-terminal Cys residues. However, the peptides were designed to facilitate penetration of the blood–brain barrier, but their interaction with the nAChRs or any other receptor was not tested. Thus, to best of our knowledge, here are the first data on the interaction of synthetic fragments of the central loop of Ly6 proteins with nAChRs.

In the case of the SLURP1 fragment, we did not manage to register the activity against α9α10 nAChR, which would be comparable to that of the synthetic SLURP1 and thus might prove useful for design of new analgesics. The aforementioned examples of the snake-venom TFPs show that, in the future, a success might come with the fragments not only of the central loop II (see **Figure 1**) but of other loops as well. In particular, mutagenesis of ws-Lynx1 demonstrated the functional role of several residues both in the central loop and in the N-terminal loop (Lyukmanova et al., 2013).

The activity of Lynx1 and SSS synthetic fragments, although being in low µM range, should not be called low. The affinity of α-neurotoxins against the muscle-type and α7 nAChRs is in the nanomolar range, but earlier in radioligand and electrophysiology experiments, it was shown that both ws-Lynx1 and recombinant SLURP1 (bearing an additional N-terminal Met residue) bind to the *Torpedo*, α7, and several heteromeric nAChRs and inhibit their currents at low µM concentrations (Lyukmanova et al., 2016b). The concentration of Lynx1 in the brain was also shown to be in the µM range (Thomsen et al., 2014). With a similar µM affinity synthetic SLURP1 (identical in the amino acid sequence to the naturally occurring protein) inhibits α9α10 and α3β2 nAChRs (but does not inhibit either rat or human α7 nAChRs). The synthetic fragment of Lynx1, at least toward muscle-type *Torpedo* nAChR, reproduces the activity of the full-size ws-Lynx1. This activity might find practical applications—for example, for design of efficient myorelaxants. In this respect, about 30-fold lower affinity of Lynx1 fragment (**1**) toward α7 nAChR can be an advantage: it resembles the case of azemiopsin, a linear peptide from the viper venom, which is considerably more selective to muscle nAChRs than to α7 nAChRs and for which preclinical studies as a myorelaxant have been recently completed (Shelukhina et al., 2018).

## Author Contributions

VT contributed to the conception and planning of the study. EK contributed to the leadership of the project, planning of experiments, and radioligand assay. NE and MZ contributed to the synthesis and characterization of peptides. IK contributed to the preparation of radioligand. DK, DL, ES, and AG contributed to the analysis of biological activities of the peptides. EK, YU, and VT contributed to the drafting of the manuscript and its editing.

## Funding

The major support of this work was by RSF grant 16-14-00215. VT was additionally supported by the RFBR grants 18-04-00844 and 17-00-00063 komfi. IK was additionally supported by the RFBR grant 18-04-01366.

## Conflict of Interest Statement

The authors declare that the research was conducted in the absence of any commercial or financial relationships that could be construed as a potential conflict of interest.

## Abbreviations

Ly6, lymphocyte antigen 6; Lynx1, Ly-6/neurotoxin; SLURP, secreted Ly6/uPAR protein; SSS, Drosophila sleepless protein; nAChR, nicotinic acetylcholine receptors; AChBP, acetylcholine-binding protein; LBD, ligand binding domain; α-Bgtx, α-bungarotoxin.
